# Postbiotic production: harnessing the power of microbial metabolites for health applications

**DOI:** 10.3389/fmicb.2023.1306192

**Published:** 2023-12-19

**Authors:** Nidhi Prajapati, Jinil Patel, Sachidanand Singh, Virendra Kumar Yadav, Chinmayi Joshi, Anil Patani, Dharmendra Prajapati, Dipak Kumar Sahoo, Ashish Patel

**Affiliations:** ^1^Department of Biotechnology, Smt. S. S. Patel Nootan Science and Commerce College, Sankalchand Patel University, Visnagar, Gujarat, India; ^2^Department of Microbiology, Smt. S. S. Patel Nootan Science and Commerce College, Sankalchand Patel University, Visnagar, Gujarat, India; ^3^Department of Biotechnology, School of Energy and Technology, Pandit Deendayal Energy University, Knowledge Corridor, Gandhinagar, Gujarat, India; ^4^Department of Life Sciences, Hemchandracharya North Gujarat University, Patan, Gujarat, India; ^5^Department of Veterinary Clinical Sciences, College of Veterinary Medicine, Iowa State University, Ames, IA, United States

**Keywords:** postbiotics, microbial metabolites, health applications, probiotics, antimicrobial activity

## Abstract

Postbiotics, which are bioactive substances derived from the metabolic processes of beneficial microbes, have received considerable attention in the field of microbiome science in recent years, presenting a promising path for exploration and innovation. This comprehensive analysis looks into the multidimensional terrain of postbiotic production, including an extensive examination of diverse postbiotic classes, revealing their sophisticated mechanisms of action and highlighting future applications that might significantly affect human health. The authors thoroughly investigate the various mechanisms that support postbiotic production, ranging from conventional fermentation procedures to cutting-edge enzyme conversion and synthetic biology approaches. The review, as an acknowledgment of the field’s developing nature, not only highlights current achievements but also navigates through the problems inherent in postbiotic production. In order to successfully include postbiotics in therapeutic interventions and the production of functional food ingredients, emphasis is given to critical elements, including improving yields, bolstering stability, and assuring safety. The knowledge presented herein sheds light on the expanding field of postbiotics and their potential to revolutionize the development of novel therapeutics and functional food ingredients.

## 1 Introduction

**Figure 1:**
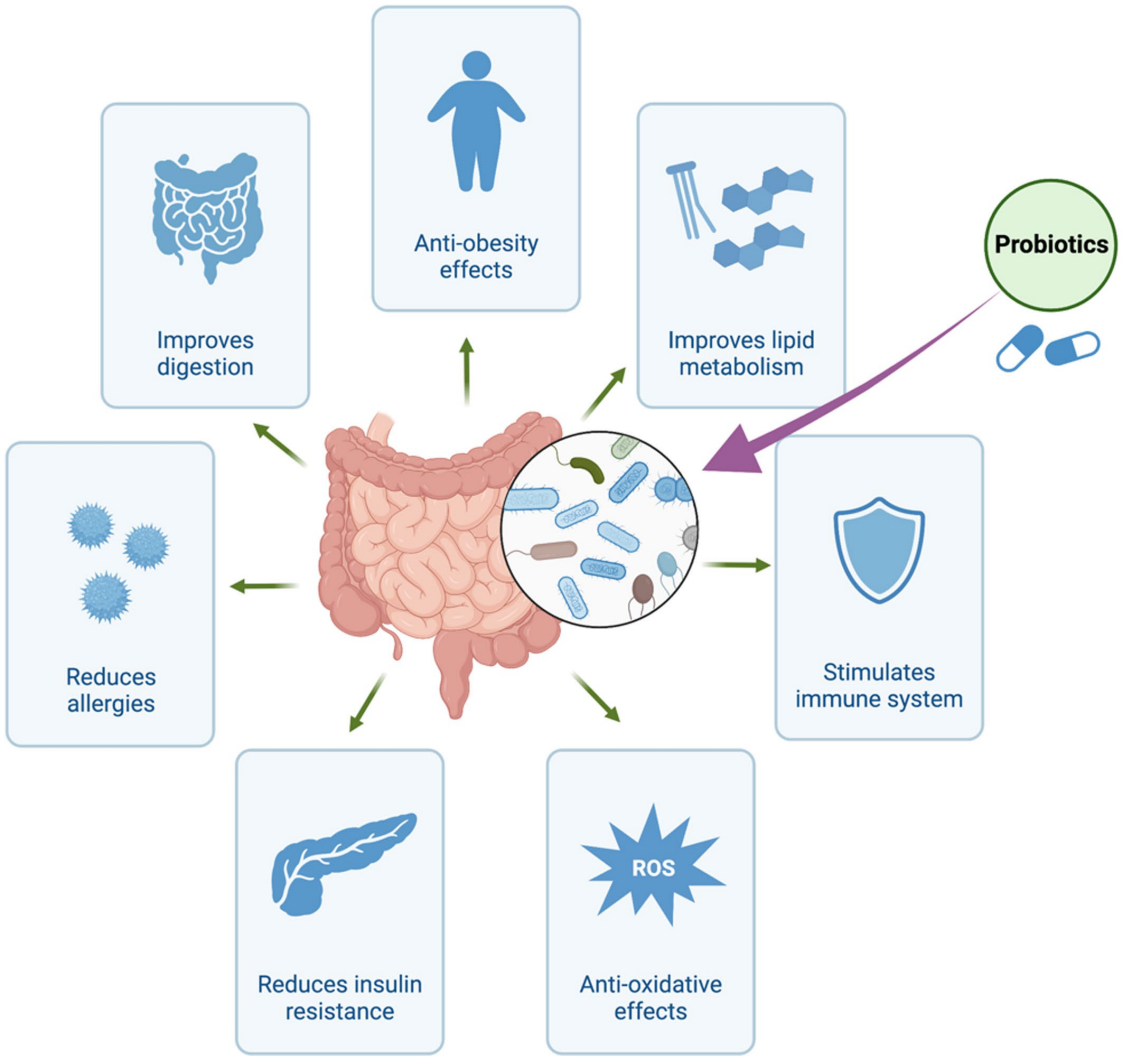
Health Benefits of Probiotics. The figure was produced with BioRender (www.biorender.com; accessed 30 July 2023).

Recent years have seen a paradigm shift in how we perceive the intricate connection between the human body and the vast community of bacteria that resides within it (Dekaboruah et al., 2020). Particularly, the human gut has developed into an intricate ecosystem home to trillions of bacteria essential to preserving our health and well-being (Kho and Lal, 2018). Exploring the therapeutic capabilities of these microorganisms has garnered significant attention due to the recognition of their ability to impact diverse physiological processes (Thursby and Juge, 2017). Traditionally, the primary emphasis has been placed on the direct application of live beneficial bacteria, commonly referred to as probiotics, to present health-promoting effects (Figure 1; Amara and Shibl, 2015; Kerry et al., 2018; Plaza-Diaz et al., 2019; Zommiti et al., 2020). However, emerging evidence suggests that the beneficial effects attributed to probiotics may not solely arise from the presence of viable microbes themselves. Rather, it appears that a significant portion of their therapeutic impact can be attributed to the metabolites they produce, referred to as postbiotics (Scott et al., 2022). Postbiotics refer to bioactive compounds produced as a result of the fermentation process of probiotics or through the metabolic activity of beneficial microorganisms residing in the gastrointestinal tract (Patani et al., 2023a). Organic acids, peptides, enzymes, and other metabolites with potential health advantages are examples of these molecules. In contrast to prebiotics and probiotics, postbiotics are characterized by their extended shelf life and enhanced stability due to the absence of living microorganisms. They have the potential to confer numerous advantages to the host, encompassing the regulation of the immune system, enhancement of gut barrier function, and promotion of overall gastrointestinal wellness (Sittipo et al., 2019). Postbiotics, predominantly derived from yeast and lactic acid bacteria, are primarily synthesized through fermentation processes (Wegh et al., 2019).

The concept of harnessing the power of postbiotic production represents a paradigm shift in microbial-based therapeutics. By redirecting attention from live microbes to the metabolites they produce, researchers can delve into a plethora of innovative health implications that transcend the constraints commonly associated with traditional probiotic approaches (Scott et al., 2022). This exciting field holds tremendous promise for developing targeted interventions to combat various diseases and enhance human health (Vinderola et al., 2022a).

In this scholarly review article, our objective is to provide a comprehensive assessment of the present status of postbiotic production and its prospective implications for health-related applications. Additionally, the intricacies of postbiotic synthesis mechanisms, the various factors that influence their production, and a variety of health benefits associated with their administration were presented. Furthermore, the research topic also addressed the challenges and future directions in developing postbiotic-based therapies, including strategies for optimizing production, formulation, and delivery. Overall, this review paper seeks to shed light on the emerging field of postbiotic production and highlight its potential to revolutionize the way we approach microbial-based therapeutics. By harnessing the power of microbial metabolites, there exists a promising opportunity to explore new avenues for improving human health, expanding our understanding of the intricate host–microbe interactions, and ultimately paving the way for innovative and personalized approaches to disease prevention and treatment.

## 2 Classes of postbiotics

**Figure 2:**
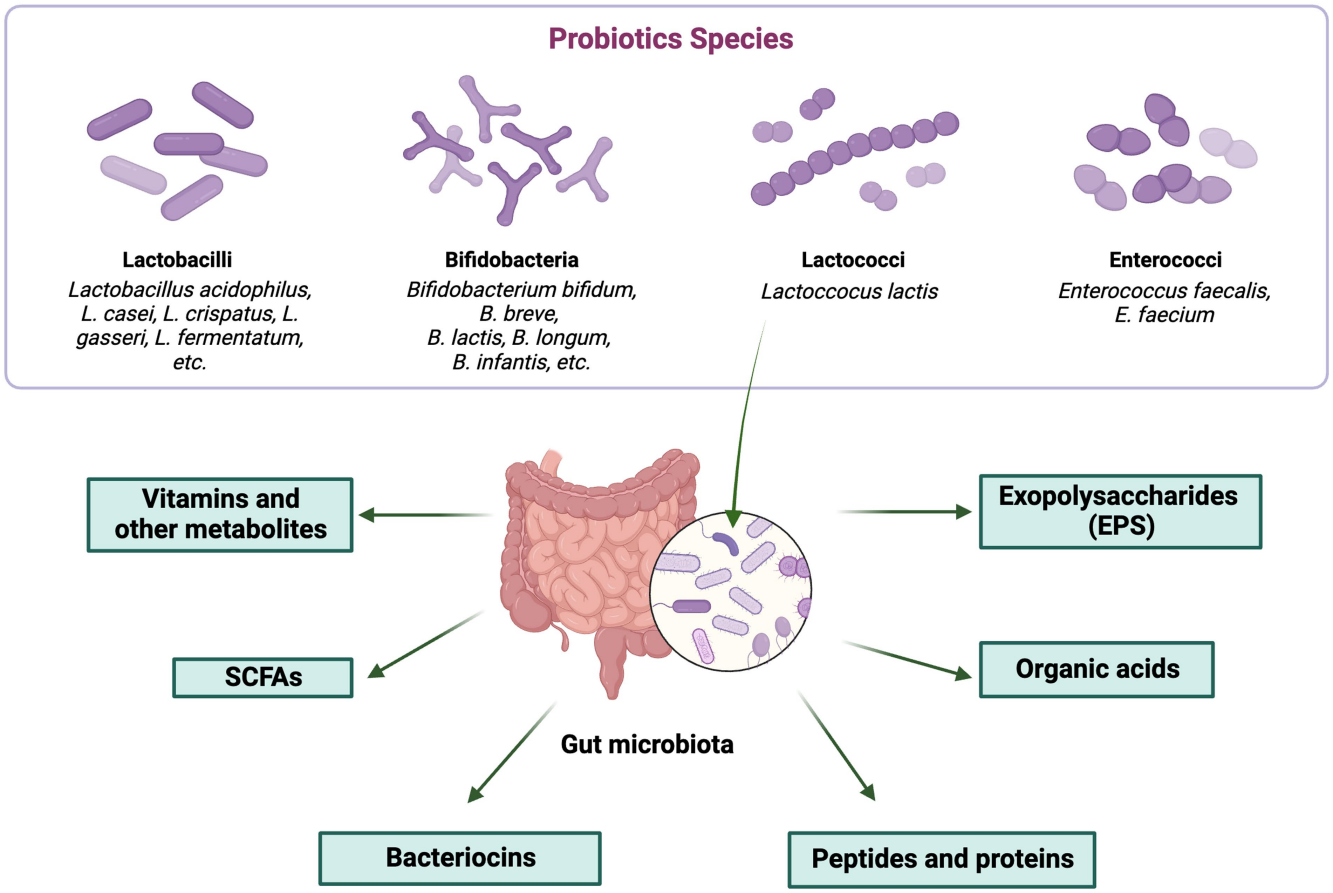
Role of probiotics in beneficial postbiotic production. The figure was produced with BioRender (www.biorender.com; accessed 27 September 2023).

The several ways in which postbiotics contribute to an improvement in general health are highlighted by the various classes. The role of probiotics in beneficial postbiotic production is depicted in Figure 2.

### 2.1 Short-chain fatty acids

**Figure 3:**
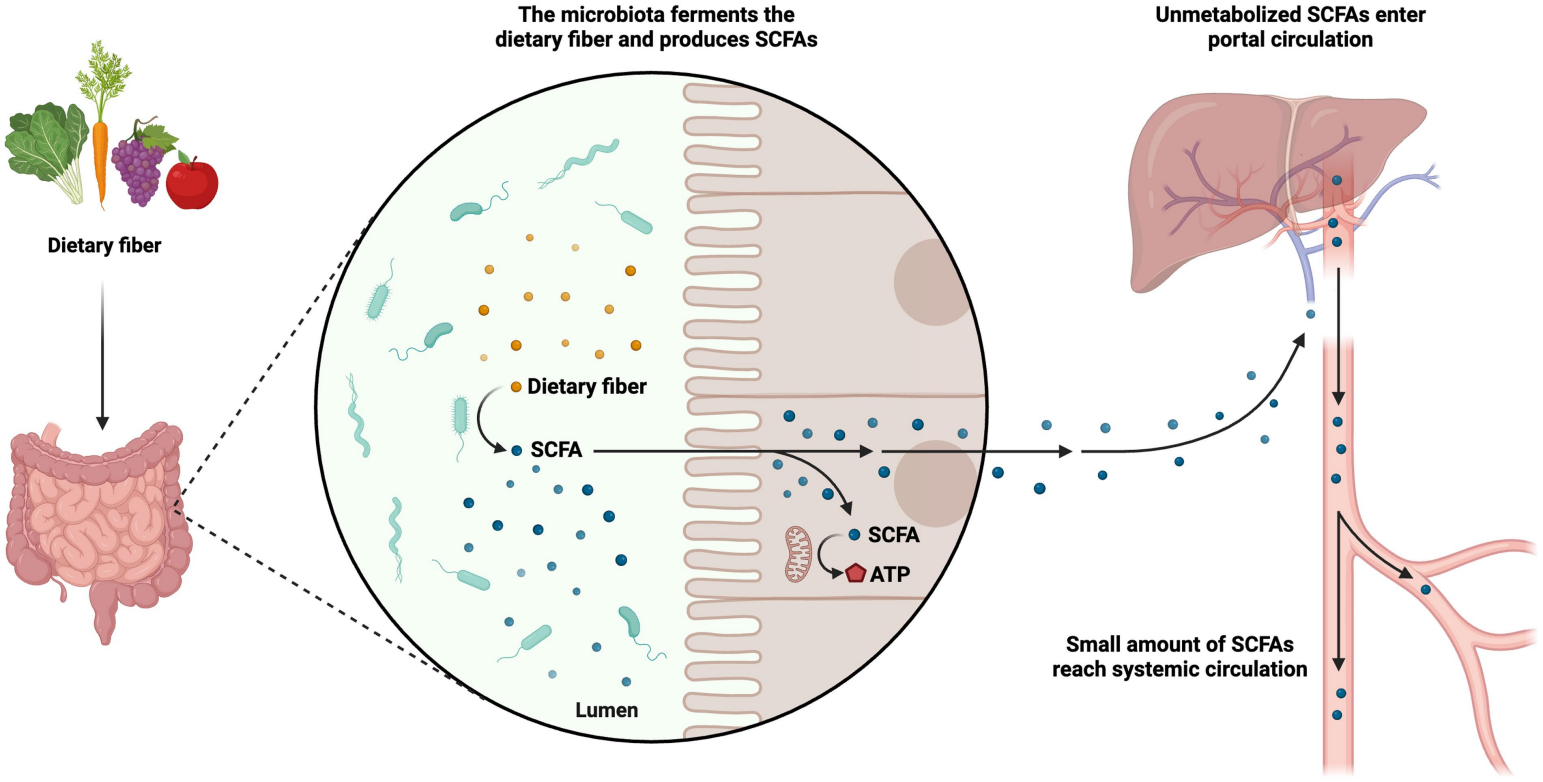
Metabolism of short-chain fatty acids (SCFAs). The metabolic pathways governing the use of SCFAs are responsible for the degradation and subsequent utilization of SCFAs within the biological system for energy production or participation in the biosynthesis of essential biomolecules. The figure was produced with BioRender (www.biorender.com; accessed 24 September 2023).

A vital class of metabolites gut bacteria make from the fermentation of plant polysaccharides is Short-chain fatty acids (SCFAs; Thorakkattu et al., 2022). Local concentrations can approach millimolar levels for these saturated aliphatic organic acids, which have a carbon backbone ranging from one to six. The gut microbiota produces SCFAs entirely from starch. Through intestinal microbial fermentation, which mostly takes place in the colon, dietary fiber, including non-digestible carbohydrates that evade absorption and digestion in the small intestine, is transformed into SCFAs. Additionally, SCFA can be produced from non-digested proteins or peptides as a substrate. The synthesis of lipids or glucose can also be accomplished using SCFAs. Thus, SCFAs produced by gut microbes give host cells, like colonocytes, access to extra energy (Sittipo et al., 2019). Particularly, the fermentation of prebiotics such as fructooligosaccharides and inulin results in the development of the SCFA propionate, acetate, and butyrate. These are present in the colon and feces in approximately 60:20:20 molar ratio (Takagi et al., 2016). Other microorganisms utilize acetate as a growth agent, and it also has a role in controlling cholesterol. The major energy sources for epithelial cells and colonocytes (Rowland et al., 2017), propionate and butyrate, play a role in gluconeogenesis and encourage the death of colon malignant cells by promoting apoptosis. *Lacticaseibacillus paracasei* ATCC 335, *Limosilactobacillus fermentum*, *Lactobacillus acidophilus*, and *Levilactobacillus brevis* produce SCFAs (Higashi et al., 2020). Studies of the relationships between postbiotics, hosts, and gut microbiota can benefit from using SCFAs in clinical trials as well as more comprehensive methodologies (Aggarwal et al., 2022). The metabolic pathways governing the use of SCFAs encompass a series of intricate enzymatic reactions. These processes are responsible for the degradation and subsequent utilization of SCFAs within the biological system for energy production or participate in the biosynthesis of essential biomolecules. The metabolic processes of SCFAs are of paramount importance in regulating energy homeostasis and preserving overall metabolic well-being (Figure 3).

### 2.2 Peptides and proteins

Bacteria play a pivotal role in the intricate process of synthesizing and generating a wide variety of peptides. Antimicrobial peptides employ pleiotropic mechanisms, such as inhibiting macromolecular synthesis and degrading microbial membranes, to effectively eliminate bacterial pathogens (Waghu and Idicula-Thomas, 2020). Ribosomal and non-ribosomal kinds of antimicrobial peptides are distinguished. By rupturing microbial membranes, ribosomal proteins produced by the bacteria exhibit potent antibacterial action *in vitro* (Makarova et al., 2019). In all bacteria, peptides are frequently found. The cell membrane is the primary target of some peptides, as was already established, whereas the cytoplasm and sensitive bacterial structures are the primary targets of other peptides. The peptides’ antimicrobial mechanisms include (a) bringing about the acidification of the bacterial membrane of cells, (b) producing physical holes that allow cells to leak out, (c) inducing processes that are fatal, such as the hydrolases, which have negative impacts on the cell wall, and (d) damaging the bacteria’ delicate internal components. *Bacillus subtilis* is one type of bacteria whose biological processes produce peptides (Rad et al., 2021). Bacteriocins refer to antimicrobial peptides synthesized by ribosomes and possess either bactericidal or bacteriostatic properties, specifically targeting bacterial strains that are similar or closely related (Wegh et al., 2019). Understanding the active molecular mechanism of probiotics depends on the proteome of the bacterial cell surface. The bacterial surface proteins are divided into four categories: (a) hydrophobic trans-membrane domains that anchor the proteins to the cytoplasmic membrane, (b) lipoproteins that are covalently bound to the membrane lipids following the cleavage of signal peptide by signal peptidase II, (c) Sortases covalently bind to peptidoglycan to form proteins having a C-terminal LPXTG-like motif. There have been reports of surface proteins from probiotic bacteria showing anti-inflammatory and anti-adhesion properties, as well as biosorption of harmful heavy metals and strengthening the epithelial barrier (Nataraj et al., 2020). For example, the presence of both cells and extractable surface proteins (S-layer proteins) from *Enterococcus faecium* WEFA23 resulted in a significant reduction in the apoptosis of Caco-2 cells that was induced by *Listeria monocytogenes* and mediated by the activation of caspase-3 (He Y. et al., 2019; Nataraj et al., 2020).

### 2.3 Bacteriocins

**Figure 4:**
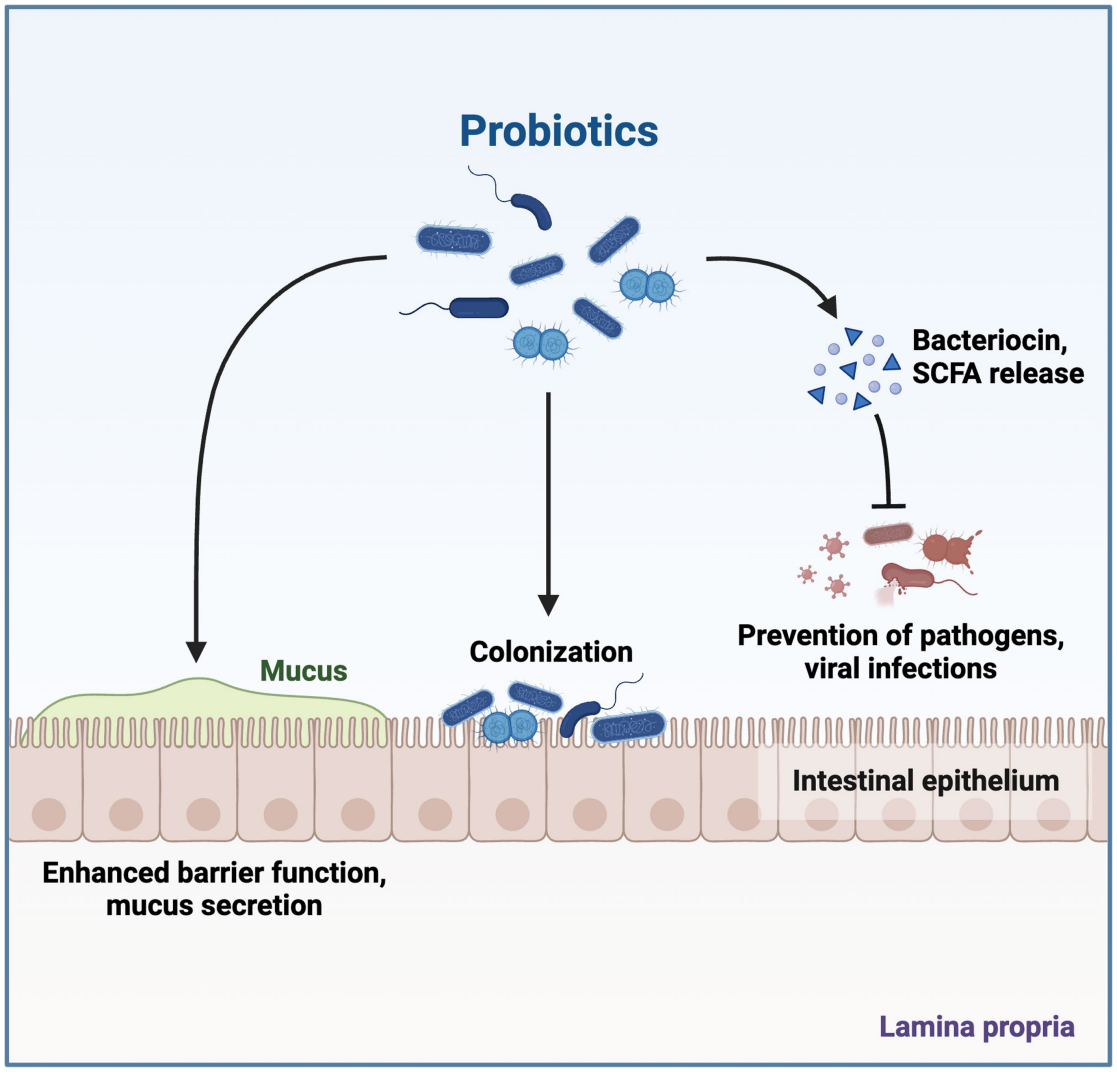
Effects of Probiotic Gut Microbiota. Postbiotics produced by probiotic microorganisms contribute to health-promoting aspects, which include enhancing the barrier against infection and also due to exhibiting antibacterial, immune-modulating, and anti-inflammatory properties. The figure was produced with BioRender (www.biorender.com; accessed 22 September 2023).

Bacteriocins, which include the peptides or proteins having antimicrobial activity, are produced by a variety of bacteria, including *Archaebacteri*a and *Eubacteria*. Humans have employed bacteriocins in fermented foods for thousands of years due to their potent antibacterial properties (O'Connor et al., 2020). Size, mode of action, and inhibitory spectrum are used to categorize bacteriocins. The growth and development of gastrointestinal infections are inhibited by bacteriocins, which also have other advantageous properties, such as resistance to heat and pH (Figure 4; Rad et al., 2021). The three main biofilm defenses these postbiotic uses are as follows: (a) inhibition of twitching motility; this ability of biofilm is mediated by pili, whereas swimming and swarming are the results of flagella activity; (b) interference with quorum sensing (QS); it affects cell interactions, colonization, and loss of QS signals; (c) reduction of virulence factors (as pyocyanin, protease, and rhamnolipid); Pyocyanin aids in the development of biofilms and revealing infection, and rhamnolipid from *Pseudomonas aeruginosa* (Lee et al., 2020). *L. acidophilus* ATCC 4356 generated bacteriocins that prevented *B. subtilis* BM19 from adhering to surfaces and forming biofilms (Moradi et al., 2020). Furthermore, they demonstrate both narrow-and wide-ranging inhibitory effectiveness against bacterial growth, attracting interest for their potential therapeutic application as next-generation antimicrobials in reducing the threat of an infectious disease caused by drug-resistant pathogens (Soltani et al., 2021). The antiviral and anticancer properties of bacteriocins are also said to exist. Even though they are odorless, colorless, and biodegradable, further study is needed to determine their safety, toxicity, and immunogenicity due to their broad application in food, medicine, cosmetics, cancer therapy, and veterinary use (Aggarwal et al., 2022).

### 2.4 Exopolysaccharides

Bacterial growth produces exopolysaccharides (EPS), which have long chains and an enormous molecular mass (Wang et al., 2020). In the production of dairy and fermented foods, such as milk, curd, sour cream, yogurt, cheese, and buttermilk, EPS derived from lactic acid bacteria (*Lactococcus*, *Leuconostoc*, *Streptococcus*, *Pediococcus*, and *Bifidobacteria*) is more frequently used to improve the flavor, taste, texture, and shelf life of fermented foods (Roca et al., 2015). Probiotic bacteria (*Leuconostoc*, *Lactobacillus*, *Lactococcus*, *Streptococcus*, *Bifidobacterium*, and *Enterococcus*) have mostly been employed to synthesize EPSs for a variety of uses (Hussain et al., 2017). The bacterial surface interactions and communication that are essential for biofilm adhesion, formation, and confirmation are mediated by these extracellular polymers. The different sugars included in EPS produced by *L. fermentum* strains are composed of different molar ratios of galactose, glucose, arabinose, and mannose. It is noteworthy to notice that EPS generated by *L. acidophilus* affected gram-negative as well as gram-positive bacteria. On the surface of the *Escherichia coli* O157:H7 biofilm, antibiofilm activity ranged from 87% to 94% (Moradi et al., 2020). By preventing cholesterol absorption, EPSs may also have a beneficial impact on lipid metabolism. In a preclinical animal (rabbit) model, kefiran intake (an EPS made by *L. kefiranofaciens*) indeed exhibited a delay in the initiation of atherosclerosis. Kefiran also reduced blood pressure and controlled blood sugar levels in rats with too much cholesterol. As a result, EPSs like kefiran are possible options for avoiding cardiovascular disorders. The Dectin-1 receptors on the surface of macrophages can bind with and activate β-glucans, another type of EPS. Consequently, β-glucans may enhance the cellular immune response to infections, including bacteria and viruses, parasites, and cancerous cells. The effectiveness of probiotics may also be enhanced by β-glucans, for instance, by promoting *Lactobacilli*’s adherence to the intestinal epithelium. They can also improve a substance called carotenoids’ bioavailability and absorption in the digestive system. Carotenoids are substances with anti-inflammatory and antioxidant capabilities (Morifuji et al., 2020). Although the precise biological significance of EPSs remains somewhat elusive, the food industry currently employs them for their water-binding, emulsifying, and stabilizing properties (Singh and Saini, 2017). However, in recent times, there has been a notable surge in the level of interest surrounding the utilization of EPSs in the domains of pharmaceuticals and functional food (Żółkiewicz et al., 2020). The *Lactobacillus* strains derived from fermented durian fruit exhibit noteworthy antimicrobial and antioxidant characteristics because of the production of various EPSs (Khalil et al., 2018). An instance of EPS derived from *L. helveticus* has been identified as uronic acid, a compound exhibiting noteworthy antioxidant characteristics in green tea (Li et al., 2014). EPSs have the potential to exert a regulatory influence on the immune response through their interactions with key immune cells such as dendritic cells and macrophages. Additionally, EPSs have been observed to augment the proliferation of T and NK lymphocytes (Makino et al., 2016), thereby further contributing to their immunomodulatory effects. An EPS derived from tofu, a byproduct of *L. plantarum*, enhances IgA concentrations within the intestinal mucosa and stimulates lymphocyte proliferation (Wang et al., 2018). EPSs may potentially exert a favorable impact on lipid metabolism through their ability to impede cholesterol absorption (Khalil et al., 2018).

### 2.5 Organic acids

Organic acids are substances that are suitable for use as antibacterial agents. One of the most important postbiotics is known as organic acids. Two isomers of lactic acid, L and D, are accessible and efficiently suppress pathogenicity when created by bacterial fermentation processes (Rad et al., 2021). Organic acids act as an acidifier, reducing the pH of the surrounding environment and the ability of infections that are not acid-tolerant to survive. Acetic and lactic acids are created to encourage the development of producer cells during the generation of postbiotics by *L. plantarum* (Chang et al., 2021). Low pH and high amounts of organic acids inhibit the growth of organisms that cause food spoilage and illnesses. Furthermore, organic acids may prevent pathogens’ enzymes from working properly, making the bacterial cell expend all its energy to release extra proton H^+^, which causes the bacteria to die (Kareem et al., 2014). This method of biopreservation, which includes combining several organic acids, could be used to generate new antibacterial agents for widespread usage in the food industry (Rad et al., 2021).

### 2.6 Vitamins and other metabolites

Vitamins are chemical substances added to food in trace amounts to support a variety of bodily biological processes. The majority of the B-complex vitamins function as coenzymes in a variety of energy metabolism reactions. The sole coenzyme-active vitamin is vitamin K, which is only fat-soluble. The majority of vitamins cannot be biosynthesized by humans; hence, they must be supplemented exogenously. Vitamins A, D, E, and others need to be added to the diet as supplements, although other vitamins, such as riboflavin (vitamin B2) and folic acid (vitamin B9), are even generated by commensal bacteria in the stomach and some probiotic microorganisms. B-group vitamins, commonly present in a wide range of foods, undergo rapid degradation when subjected to thermal processing. *Propionibacterium freudenreichii* 2067 is capable of producing vitamin B12 (Nataraj et al., 2020). These vitamins are necessary for many physiological activities, including the following: Vitamin K plays a role in blood clotting as a cofactor of gamma carboxylase activity. Riboflavin plays a role in redox activities as a hydrogen transporter. Folate plays a part in DNA replication, repair, and methylation. Many of these vital vitamins are provided by gut commensal bacteria, particularly lactic acid bacteria and *Bifidobacterium* species, including riboflavin, folate, cobalamin, thymine, pyridoxine, nicotinic acid, and niacin (Aggarwal et al., 2022). Since they are crucial for generating energy, regulating genes, and modifying intestinal immunity, vitamin B is known to have anticancer characteristics. The water-soluble vitamin cobalamin, or vitamin B12 (B12), is crucial for sustaining the health of neurons and hematopoiesis. It is a nutrient that is present in animal products. The health of bones and the circulatory system has been demonstrated to benefit from vitamin K (Thorakkattu et al., 2022).

## 3 Mechanisms of action

### 3.1 Immunomodulation

The gut microbiome’s immunomodulatory properties have long been postulated. Regulatory T cells (Tregs) are induced to differentiate in the gut by butyrate, a SCFA. Moreover, propionate (another SCFA) promotes the development of peripheral Tregs. In addition to producing anti-inflammatory cytokines and fostering T helper (Th)2-dependent immune responses, several postbiotic fractions (supernatant, cell wall fragments) recovered from *B. coagulans* culture also do so. Additionally, *in vitro* studies have shown that the supernatant of a *Bifidobacterium breve* culture improves dendritic cell survival and maturation, which in turn increases IL-10 production and reduces TNF-α release. As seen in individuals who are prone to atopic disorders, these characteristics may be to blame for reducing Th1-mediated responses while enhancing Th2-mediated responses (Ruiz et al., 2017; Żółkiewicz et al., 2020; Hickey et al., 2021). Making the body resistant to viral infection begins with immune system modification. Numerous clinical investigations have examined the direct and intricate connection between probiotics, ideal immune system performance, and homeostatic circumstances (Pacini and Ruggiero, 2017; Valdes et al., 2018; Mazziotta et al., 2023). Regarding postbiotics’ biological efficacy in decreasing inflammatory reactions, postbiotics demonstrate a great capacity to do so when compared to inflammatory reactions brought on by substances like lipopolysaccharide (McCabe and Parameswaran, 2018). In order to change the immune system, the postbiotic substance primarily affects CD8+ T cells, interferon-gamma (IFN-γ), granulocyte phagocytic activity, tumor necrosis factor-alpha (TNF-α), proinflammatory cytokines, and elevated levels of IL-10 and IL-4 cytokines. The importance of microbial species, inactivation techniques, and an efficient postbiotic maintenance dose in delivery systems should be emphasized (Khani et al., 2022). By creating the anti-inflammatory cytokine IL-10 *in vitro* utilizing *L. reuteri* 17,938, retinoic acid-driven mucosal-like dendritic cells have an impact on immunomodulation (Aguilar-Toalá et al., 2018). SCFAs, produced during bacterial fermentation, alter immune cell function by boosting anti-inflammatory responses (Nogal et al., 2021). Lipoteichoic acid and peptidoglycan, components of microbial cells, trigger immunological receptors, regulating cytokine production (Szentirmai et al., 2021). Bacterial activity produces metabolites such as lactate and acetate, which can alter T-cell differentiation and function (Wojciech et al., 2020). Postbiotics also affect mucosal immunity by raising secretory IgA production (Szydłowska and Sionek, 2022).

### 3.2 Anti-inflammatory effects

Intense illnesses are identified by lymphopenia, multiple organ failure, excessive inflammation, acute respiratory distress syndrome (ARDS), and high fatality rates. The over-formation of inflammation in illnesses is made possible by intestinal dysbiosis, which also results in chronic inflammation and diminished anti-inflammatory mechanisms. Through its outstanding anti-inflammatory efficacy, postbiotics might lessen the severity of illnesses. Postbiotics also block a variety of proinflammatory signaling pathways to lessen cytokine storms (Khani et al., 2022). Clinical research on the anti-inflammatory properties of the *B. longum* CECT-7347 strain of postbiotics revealed that they were successful in reducing gastrointestinal disturbance and acute inflammatory response as well as by activating the immunological pathways linked to a specific immune response (Thorakkattu et al., 2022). Additionally, human colorectal cancer cell lines triggered by LPS produce less IL-8, which suggests that compound K has anti-inflammatory properties. Most metabolites act anti-inflammatory together, which is important for tissue homeostasis. High amounts of metabolites, however, may result in adverse host reactions. For instance, protracted anti-inflammatory effects may raise the risk of infectious disease (Sittipo et al., 2019). Certain heat-killed bacterial strains prevent the release of IL-8 in the intestinal cells by releasing soluble anti-inflammatory proteins that can trigger cellular immunological and anti-inflammatory responses (Aggarwal et al., 2022).

### 3.3 Antimicrobial activity

Peptides, bacteriocins, organic acids, and volatile substances found in the isolates’ metabolites have been connected to postbiotics’ antibacterial properties and may also be in the role of inhibiting pathogen adhesion to the intestine (Figure 4; Tarique et al., 2022). One of the advantages of postbiotics is their ability to prevent harmful bacteria from growing and food spoiling. These substances are frequently used nowadays to combat pathogenic bacteria and food spoiling because postbiotics have advantages over antibiotics and artificial preservatives. The type of target bacterium (Gram-positives are more resistant to postbiotic compounds than gram-negatives), the type of probiotic from which the postbiotic is prepared, and the concentration of postbiotics all affect how antibacterial a postbiotic is (Moradi et al., 2020). Because they aid in sealing the intestinal barrier and competitively bind to pathogen-necessary receptors, several postbiotics may have direct antimicrobial effects (Nataraj et al., 2020). Thus, they alter the expression of genes in the host or modify the environment in the area. The effects of postbiotics generated from *L. plantarum* were examined in an *in vitro* experiment. Supernatants of *Bifidobacterium* and *Lactobacillus* cultures have recently been found to exhibit antibacterial activity as well. By stopping enteroinvasive *E. coli* strains from entering enterocytes, this could be seen *in vitro*. The inhibition of hazardous bacterial adhesion (as they compete for receptor sites) may be the cause of these antibacterial properties, but the cellular supernatants might also have local impacts on the expression of resistance genes, cell barriers, and intestinal environment (Aghebati-Maleki et al., 2021). Pathogens are prevented in the stomach through competitive ejection, which depends on antimicrobial action. A typical candidate probiotic *Lactobacillus* isolates must produce antimicrobial compounds such as organic acids (acetic acid, lactic acid, and propionic acid), bacteriocin, diacetyl, H_2_O_2_, and surfactants in order to have antimicrobial activity opposite to a variety of pathogenic microorganisms (Meena et al., 2022). It has been demonstrated that postbiotics are antibacterial against both Gram-positive and Gram-negative microorganisms (Ma et al., 2023). Numerous known and unexplained antimicrobial substances, most frequently bacteriocins, enzymes, tiny molecules, and organic acids, are responsible for this antimicrobial activity (Aguilar-Toalá et al., 2018). Utilizing postbiotics to prevent food-spoilage bacteria is one of the most significant effects on the food sector. Postbiotics exhibit notable antimicrobial properties due to the presence of various bioactive compounds such as peptides, organic acids, fatty acids, bacteriocins, and hydrogen peroxide molecules (Rad et al., 2021).

### 3.4 Gut barrier function

The gut microbiota produces various compounds, including vitamins, metabolites from phenols, and aromatic amino acids. The catabolism of aromatic amino acids (AAAs) by the gut microbiome results in the production of a variety of metabolites that have the potential to modulate immune, metabolic, and neuronal responses. Due to their biological activity, AAA molecules can potentially affect distant organs like the kidneys, brain, and cardiovascular system. Genetic changes in gut microbial metabolism, for example, can affect indoxyl sulfate plasma levels. The presence of indoxyl sulfate has been found to have a significant role in the advancement of chronic kidney disease, suggesting that modulating AAA metabolism may have potential therapeutic implications for renal illnesses (Żółkiewicz et al., 2020). Postbiotics have the potential to exert a discernible influence on the composition and functionality of the gut microbiota. The inhibitory effects on the growth and virulence of potential pathogens can be observed through the utilization of postbiotics, such as SCFA. The utilization of organic acids by specific bacterial species in the stomach is an additional facet of their secretory function. Postbiotics have the ability to bind to intestinal epithelial cells in this way, preventing infections from adhering to these cells. Intestinal permeability is controlled by postbiotics once they have bonded to the intestinal epithelial cells and are preventing pathogen development. Additionally, by reducing inflammation and strengthening the epithelial barrier, postbiotics help restore gut health. Postbiotics have the ability to improve intestinal barrier performance and prevent the invasion of harmful microorganisms in the initial phase. Antimicrobial substances such as defensins, SCFAs, and bacteriocins also directly impede the development of pathogens (Khani et al., 2022). The gut microbiome of an individual encompasses a diverse assemblage of bacteria comprising both beneficial and pathogenic strains and reflects familial inheritance. There is a very fine line between the two, and any disruption of this normal microflora (dysbiosis; Sahoo et al., 2022, 2023) affects not only the gastrointestinal tract (GIT) but also other organs, making them less effective (Aggarwal et al., 2022). The gut microbiome has the capacity to engage in interactions with specific immune cells as well as various types of human cells. These interactions provide the host with several health advantages, including the regulation of GIT motility, removal of toxins and mutagens, conversion of bile acid and steroids, generation of vitamins, mineral absorption, and modulation of systemic and mucosal immunity. Isolating lactic acid bacteria from fermented food products with potential probiotic effects is essential for improving the quality of the gut microbiota (Alameri et al., 2022). Aromatic amino acids are produced and metabolized by gut microbiomes, and these bioactive chemicals have an impact on the cardiovascular, renal, and nervous systems. Also produced by the gut bacteria are dietary polyphenols (Thorakkattu et al., 2022). The effects of probiotic gut microbiota are depicted in Figure 4.

### 3.5 Metabolic effects

The host experiences physiological and nutritional benefits from postbiotics, including non-viable intact microorganisms, their subcellular components, and metabolic byproducts released by living bacteria during their growth or after bacterial cell lysis (Salminen et al., 2021). The disruption of cell membranes, drop in cytosol pH, generation of hydroxyl radicals, and interference with cellular metabolic activity were all signs of organic acid’s antibacterial activity against periodontal infections. Hydrogen peroxide and released proteins also had this effect. The COVID-19 pandemic may be controlled because recent research reveals that postbiotic structure and metabolic activity might be utilized as biomarkers for anticipating viral diseases like coronavirus sickness. Many of the health benefits of pre-, pro-, and syn-biotics appeared to be mediated by a variety of metabolic byproducts, cellular and subcellular structural elements, and whole or ruptured dead microorganisms. Cell-free supernatants, teichoic acid, cell wall fragments, bacterial lysates, vitamins, short-chain fatty acids, enzymes, exopolysaccharides, amino acids, different peptides, and fermentation by-products are examples of postbiotics, which microbial compounds are the structural and metabolic products. The postbiotic components are synthesized by probiotics through various mechanisms, which include the consumption of prebiotics, prolonged storage or processing, such as pasteurization or baking, and metabolic processes undertaken by the probiotics themselves (Aggarwal et al., 2022). It is imperative to acknowledge that considerable variation exists in the gut microbiota composition across diverse populations and even among individuals. Its functional and metabolic characteristics are related to the gut microbiota’s makeup. As a result, each person may have a different level of component microbial metabolization (Wegh et al., 2019). Postbiotics can also be given in a controlled and regular way; however, when living bacteria are given, the quantity and metabolic function of the specific strain determines how much active structure will be present in the colon. Recently, cell-free formulations with the potential to be utilized as medications for treating or preventing diseases have been created using the metabolic byproducts of several beneficial bacteria (Aguilar-Toalá et al., 2018).

### 3.6 Antioxidative activity

Probiotics slow the growth of viruses and bacteria that cause food to spoil while being stored, making them a significant tool for food preservation and disease prevention. Probiotics also have potent antioxidant effects, which help to keep food from oxidizing during storage (Chetwin et al., 2019; Barros et al., 2020). Postbiotics have specific defense mechanisms that effectively reduce the harmful effects of reactive oxygen species (ROS), which may degrade nucleic acids, lipids, carbohydrates, and proteins. Antioxidant enzymes, particularly superoxide dismutase (SOD), catalase (CAT), and glutathione peroxidase (GPx), play important roles in combating reactive oxygen species (Sahoo et al., 2019; Aghebati-Maleki et al., 2021; Patel et al., 2023; Sahoo and Chainy, 2023; Patani et al., 2023b). Hydrogen peroxide (H_2_O_2_) undergoes a catalytic transformation into molecular oxygen and water through the enzymatic action of CAT, which is ubiquitously found in various biological entities, including probiotics and other living organisms (He Z. et al., 2019). CAT can be classified as a postbiotic entity due to its origin as a metabolite produced by probiotic bacteria. As a member of the reductase oxidase family, CAT is known for its role in the prevention of ROS in general and by inhibiting ROS reactivity, it functions as an endogenous antioxidant and confers cellular protection against oxidative stress (Kleniewska and Pawliczak, 2019). The metalloenzyme, SOD, facilitates the process of dismutation, wherein superoxide radicals (O_2_^−^) undergo transformation into conventional oxygen (O_2_) and hydrogen peroxide (H_2_O_2_) molecules (Lin et al., 2020; Prajapati et al., 2023). By virtue of its antioxidative properties, SOD serves as a protective agent against the harmful effects of oxidative stress on various tissues (Sahoo and Chainy, 2007) and also represents a variant of the postbiotic entity that exhibits potential utility as an antioxidant moiety (Hassaan et al., 2021). Similarly, GPx, a crucial selenoenzyme, also safeguards cells against oxidative stress by participating in various biological processes. Its primary function involves the detoxification of H_2_O_2_ and hydroperoxides through the utilization of reduced glutathione (Sahoo and Roy, 2012; Brigelius-Flohé and Flohé, 2020). The postbiotics derived from *L. plantarum* RG14 were found to enhance antioxidant activity in post-weaning lambs, as evidenced by increased GPx levels in both serum and ruminal fluid (Izuddin et al., 2020).

## 4 Approaches for postbiotic production

### 4.1 Fermentation

The strains of *Lactobacillus, Streptococcus, Bifidobacterium, Eubacterium, Saccharomyces,* and *Faecalibacterium* are the most widespread bacteria and fungi that create postbiotics. Numerous fermented foods, such as pickled vegetables, sauerkraut, yogurt, and kombucha, contain these microorganisms. Natural fermentation results in postbiotics that cannot be controlled and may not be significant enough to have an *in vivo* physiological effect. In order to enable their study and use in culinary, pharmaceutical, and nutraceutical applications, researchers have looked at manufacturing strategies to create postbiotics in a regulated and efficient manner (Thorakkattu et al., 2022).

An industrial fermentation-based strategy is becoming more common for producing postbiotics with potential health advantages. For example, studies have shown that fermentation of probiotic bacteria such as *Bifidobacterium* and *Lactobacillus* strains can effectively produce postbiotics (Thorakkattu et al., 2022). During fermentation, these microbes create a wide range of metabolites, including organic acids, peptides, and exopolysaccharides. Anti-inflammatory, antioxidant, and immunomodulatory activities have been demonstrated for these postbiotics (Rafique et al., 2023). Because industrial fermentation allows for the regulated synthesis of these bioactive chemicals, it is a viable and sustainable method for producing functional components for use in a variety of health-promoting applications. As researchers continue to investigate the complex interaction between gut microbiota and human health, the role of postbiotics produced by fermentation has been recognized as a valuable route for improving health (Wegh et al., 2019).

Izuddin et al. (2018) state that an *in vitro* experiment was conducted to examine the effects of various postbiotic inclusion levels of *L. plantarum* RG14 on the rumen fermentation patterns, gas production kinetics, and microbial population in goat rumen fluid. The co-production of bacteriocins, EPSs, and conjugated linoleic acid (CLA) by *B. lactis* BB12 in supplemented cheese whey was improved using the Box–Behnken design (Amiri et al., 2021). The role of bacteria and yeasts utilized for sourdough (SD), the development of postbiotic-like components impacted by SD fermentation and the baking process, and the implications of functional SD bread intake for human health (De Vero et al., 2021; Pérez-Alvarado et al., 2022).

### 4.2 Enzymatic conversion

Cultured celery powder may include nitrites that have already undergone nitrate reductase (NiR) conversion in order to serve as a natural source of nitrite for meat products. In slower curing processes, non-converted celery juice powder can also serve as a source of nitrate. For the microbial route to convert nitrate to nitrite, certain NiR enzyme-producing strains are required. Nitrite reduction to nitric oxide happens naturally during meat curing as a result of interactions between the various meat components and the chemicals utilized. Cured meats can be made without using sodium nitrite directly by combining a natural nitrate source with starter cultures that reduce nitrite. However, converting nitrate to nitrite requires an incubation stage that initiates the microbial enzymatic activity (Oliveira et al., 2021). *Gordonibacter urolithinfaciens,* and *Ellagibacter isourolithinifaciens* are two bacterial species identified within the human gastrointestinal tract that possess the capability to metabolize ellagic acid and produce urolithins. Different catechol-dehydroxylases are responsible for catalyzing the dehydroxylation reactions that result in the production of urolithins, which are bioactive postbiotics. Because various catechol-and double-bond-containing phenolics (resveratrol, esculetin, scoparone, and umbelliferone) were not degraded, the enzyme activities appear to have a limited substrate range (García-Villalba et al., 2020).

With a focus on examining the metabolism of carbohydrates and enzymatic function, as well as its capability to restrict pathogen growth, the metabolic profile of *L. plantarum* K16 was studied. In addition, the *L. plantarum* K16 strain had good activity for the enzyme’s valine arylamidase or cystine arylamidase, hydrolyzing 10 to 20 nmoles of the substrate. This strain’s naphthol-AS-BI-phosphohydrolase was more active, capable of hydrolyzing 20 to 30 nmoles of the substrate (Diez-Gutiérrez et al., 2022).

### 4.3 Synthetic biology

In the rapidly expanding field of synthetic biology, preprogrammed cellular behavior is attempted to be created and realized utilizing both natural and synthetic biological components. Numerous biotechnological advancements, ranging from sophisticated medicines to the biosynthesis of chemical products, have been made because of this form of forward engineering (Ullah et al., 2016). The development and fast analysis of extensive libraries of genetic components by next-generation sequencing techniques facilitated the advancement in the research related to the biosynthesis of chemical products. Synthetic cell-to-cell communication, intricate and expansive genomic circuits, and CRISPR-based regulation are noteworthy advancements (Marchand and Collins, 2016). Synthetic biology offers a way to research structure–function interactions among bacteria and create new biotic treatments in the field of microbiota engineering. The ability of the designed microorganism to perceive, record, and react to its immediate environment is expanded by incorporating synthetic genetic components (Bober et al., 2018). Synthetic biology is essential to post-biotic production because it allows for the efficient and sustainable manufacture of useful substances using living organisms or biological components. Furthermore, synthetic biology has made it possible to build biofactories where created cells manufacture medicines like insulin and vaccines, providing more affordable and scalable production techniques (Fang et al., 2020).

## 5 Challenges in postbiotic production

### 5.1 Yield optimization

The optimization of yield in postbiotic production poses a formidable challenge, as it necessitates the delicate equilibrium of intricate factors such as complex metabolic pathways, strain-specific variability, and meticulous fine-tuning of fermentation conditions in order to achieve maximum productivity (Thorakkattu et al., 2022). This endeavor includes selecting the correct probiotic strains, developing their metabolic pathways, optimizing growing conditions, and implementing efficient downstream processing procedures while considering economic viability and regulatory compliance (Fenster et al., 2019). Things become considerably more complicated when production is scaled up from the laboratory to the industrial level (Du et al., 2022). Successful yield optimization is critical for realizing postbiotics’ full potential in health and industry, fostering innovation, and assuring cost-effectiveness while maintaining product quality and regulatory standards (Wegh et al., 2019).

### 5.2 Stability and storage

Postbiotics, the metabolic byproducts or cellular components of probiotics or microorganisms employed for various health and industrial uses, present important stability and storage problems. A postbiotic product must be stored properly to preserve its effectiveness and safety. Supernatants, cell wall constituents, and extracellular metabolites are examples of postbiotics that can be sensitive to environmental elements like temperature, humidity, and light. In order to prevent microbial deterioration and maintain their bioactivity, it is essential to store them in carefully regulated conditions, often at refrigeration temperatures (2–8°C) or even freezing temperatures (−20°C or below; Vinderola et al., 2022b).

Packaging is also important for postbiotic stability. Because oxygen and moisture can hasten the breakdown of postbiotic substances, impermeable packaging materials and vacuum-sealed containers are frequently utilized to limit exposure to these elements. Additionally, postbiotic items can be protected from light-induced degradation by using opaque or UV-resistant packaging. Regular monitoring of postbiotic products during storage is required to ensure long-term stability. Stability testing entails assessing several characteristics, such as active ingredient concentration, pH, microbial contamination, and overall quality. Manufacturers use recognized stability testing techniques to assess shelf-life and storage settings that will keep postbiotic products effective (De Almeida et al., 2023).

### 5.3 Safety considerations

There has been a surge in research and entrepreneurial attention toward the utilization of microbial administration to enhance health due to the considerable potential of probiotics in generating advantageous health outcomes. There has been an increase in consumer attention toward products that promote health and well-being. Because of considerable advances in research on the interactions between food, microbiota, and host, microorganisms have been administered, or the human microbiota has been altered utilizing cutting-edge therapeutic approaches in recent years (Reid et al., 2019). Postbiotics are presumed to possess a lower risk profile compared to probiotics due to the absence of bacterial growth within them. This crucial characteristic serves as a preventive measure against the development of bacteremia or fungemia, which are two potential hazards commonly associated with probiotic interventions (Yelin et al., 2019).

However, the progenitor bacterium’s safety profile cannot guarantee the safety of postbiotics. Postbiotic regulatory guidance must, therefore, be anticipated based on potential risks and safety issues. Significant research is required to increase our understanding of the key problems of postbiotic safety, both on a small scale in the lab and in animal models and clinical trials (Thorakkattu et al., 2022). Because of the increased need for food and feed as a result of modern countries’ growing human populations, the quality and safety of food and feed products are critical for societal health and well-being. Given the possible risks, regulatory advice and safety concerns regarding postbiotics and associated functional foods should be anticipated. The most of the probiotic foods contain *Lactobacillus* and/or *Bifidobacterium* species, which are rarely known to cause clinical diseases in humans. Despite all of the risks associated with foodborne bacteria, some helpful bacteria, such as *Lactobacillus* and *Bifidobacteria*, can outperform pathogenic bacteria and generate antimicrobial agents, which increase food safety and shelf life (Moradi et al., 2020). Because postbiotics are safe, they should be used in the food and pharmaceutical industries (Aghebati-Maleki et al., 2021).

## 6 Applications of postbiotics

Postbiotics provide a diverse range of benefits for overall well-being, from improving gut immunity and digestion to demonstrating potential anti-inflammatory characteristics (da Silva Vale et al., 2023). Table 1 shows postbiotic-producing organisms and their applications.

**Table 1:** Postbiotic-producing organisms and their applications.

Sl. no.	Postbiotic producing organisms	Name of postbiotics	Applications	References
1	*B. coagulans MTCC 5856*	Cell-Free Supernatants	Formulation of a cream containing (LactoSporin) for the treatment of Acne vulgaris	Majeed et al. (2020)
2	*L. kunkei*	Bioactive peptides (PlnA)	Topical application for 3 months of a product containing a postbiotic resulted in a significant improvement in patients diagnosed with alopecia	Rinaldi et al. (2020)
3	*Bifidobacterium breve C50 and Streptococcus thermophilus*	Thermally non-viable	Lower abdominal distention Improved inflammatory and immune markers	Campeotto et al. (2011)
4	*L. delbrueckii subsp. bulgaricus, Lactobacillus thermophilus*	Cell free supernatant	Modulation of gut microbiota	Zeng et al. (2016)
5	*L. casei, L. rhamnosus GG*	Thermally non-viable and Cell free supernatant	Anti-inflammatory	Wang et al. (2013) and Cicenia et al. (2015)
6	*Lacticaseibacillus paracasei K71*	Thermally non-viable	Anti-allergic	Moroi et al. (2011)
7	*L. plantarum L-14*	Exopolysaccharide	Reduction of obesity	Lee et al. (2021)
8	*L. brevis*	Long chain polyphosphate	Colitis with metabolic disorder	Isozaki et al. (2021)
9	*L. plantarum 70,810, L. casei*	Fermentation products	Anti-tumor	Wang et al. (2014) and Fichera et al. (2016)
10	*L. paracasei D3-5 strain*	LTA (lipoteichoic acids)	Anti-aging	Wang et al. (2020)
11	*Lactobacillus* spp.	Heat killed cells	Anti-biofilm effect against oral pathogens	Ciandrini et al. (2017)
12	*L. rhamnosus GR-1*	Cell-free supernatant	Immunomodulatory activity	Koscik et al. (2018)
13	*L. delbrueckii subsp. Bulgaricus*	EPS	Cholesterol-lowering effect	Tok and Aslim (2010)
14	*L. gasseri*	Biosurfactants	Antibiofilm ability against methicillin-resistant *S. aureus* (MRSA)	Giordani et al. (2019)
15	*L. plantarum*	LTA (lipoteichoic acids)	Antibiofilm activity against *S. mutans*	Ahn et al. (2018)
16	*L. paracasei*	Peptidoglycan	Anti-tumor effect	Fichera et al. (2016)
17	*L. kefiranofaciens*	Surface layer proteins	Melioration of *Clostridium difficile* induced cytotoxicity	Carasi et al. (2012)
18	*L. acidophilus* DDS-1	SCFA	Increases in short-chain fatty acids (butyrate, propionate, and acetate) levels	Vemuri et al. (2019)
19	*L. rhamnosus* strain ASCC 1520	SCFA	Gut microbiota alteration	Dai et al. (2019)
20	*B. bifidum*	SCFA (acetate)	Increase in TEER values	Hsieh et al. (2015)

### 6.1 Therapeutics

There is evidence that postbiotics are beneficial for health, including localized effects on specific gut epithelial tissues with immunomodulatory, anti-inflammatory, and antibacterial properties, as well as systemic effects by affecting numerous organs or tissues with anticarcinogenic and antiproliferative benefits as well as the prevention of celiac disease (Malashree et al., 2019). Therefore, there is an imminent necessity for undertaking postbiotics research in humans, alongside the imperative verification of the therapeutic and non-therapeutic impacts of these bioactive compounds. Novel postbiotic formulations could pave the way for innovative therapeutic and preventative clinical strategies in a variety of fields, including diabetes, wound healing, adjunctive therapeutic medications, food biopreservation, food packaging, biofilm management, functional food, food supplements, and pharmaceutical food. Using cutting-edge technology, studies are being done to identify and isolate various postbiotic components as well as their bioactivities for potential therapeutic applications in the future of medicine (Aggarwal et al., 2022). These bioactive substances have shown promise in treating various medical problems, including inflammatory bowel disease, irritable bowel syndrome, and allergies (Hagan et al., 2021). Furthermore, postbiotics may help to maintain a healthy gut flora, boosting overall digestive health. Because postbiotics are non-viable, they are a safer choice for people with impaired immune systems who may not be candidates for live probiotics (Wegh et al., 2019). The therapeutic effects of *L. paracasei* derived postbiotics on colitis in mice were discovered, with a significant reduction in inflammatory markers and an increase in overall gut health. As research on postbiotics progresses, the therapeutic applications of postbiotics are expected to broaden, offering new possibilities for developing therapies aimed at enhancing human health (Aggarwal et al., 2022).

### 6.2 Functional food and beverages

According to research, postbiotics include anti-inflammatory, antioxidant, and immunomodulatory properties, contributing to gut health and overall well-being (Żółkiewicz et al., 2020). A recent study, for example, looked at the postbiotic potential of *L. plantarum* metabolites, finding that they can boost the production of short-chain fatty acids, which are known to support intestinal epithelial integrity (Markowiak-Kopeć and Śliżewska, 2020). Furthermore, research on metabolites produced from *B. breve* exhibited potential in controlling glucose metabolism (Ohno et al., 2022). Integrating postbiotics into functional foods and beverages, such as yogurt, fermented drinks, or even snacks, is an innovative strategy for delivering these health advantages in a practical and pleasant way (Thorakkattu et al., 2022). As our understanding of postbiotics grows, their inclusion into ordinary food choices has enormous potential for boosting gut health and preventing a variety of chronic diseases (Wegh et al., 2019).

### 6.3 Personal care products

The skin microbiome is comprised of a diverse assemblage of fungi, bacteria, and viruses, collectively forming a complex population. Maintaining skin commensal bacteria is critical for preventing the growth of pathogenic microbes or opportunistic pathogens that are already present (Skowron et al., 2021). Consequently, developing bioactive compounds capable of controlling the skin’s microbiota has gained significance among scientists and the cosmetics industry (Nicholas-Haizelden et al., 2023). Scientific evidence is mounting that metabolites generated from probiotics have a strong potential to prevent skin disorders (da Silva Vale et al., 2023). According to scientific data, utilizing topical probiotics can lower the number of bacteria that cause acne, including *Staphylococcus epidermidis*, *S. aureus*, *Streptococcus pyogenes*, and *Cutibacterium acnes* (Aggarwal et al., 2022). Recently, there has been a significant amount of focus directed toward the investigation of probiotics and postbiotics for skincare, as these organic products have garnered recognition for their remarkable effectiveness (Zhang et al., 2022).

### 6.4 Agriculture and animal health

Postbiotics emerged as promising agents for improving agricultural practices and benefiting animal health (Thorakkattu et al., 2022). Postbiotics are a sustainable and environmentally friendly way to improve soil health and crop productivity in agriculture. These substances have the ability to increase nutrient availability, control diseases, and improve plant growth (Vassileva et al., 2020). Postbiotics contribute to crop resilience against various environmental stressors, such as drought and disease, by promoting a balanced soil microbial community (Fiodor et al., 2021). Furthermore, the application of postbiotics in agriculture aligns with the increased desire for organic and sustainable farming practices (Aggarwal et al., 2022).

Postbiotics have demonstrated significant promise for improving gut health and overall wellness in animal health. Postbiotics provide advantages for livestock, in particular when they are fed. These substances help regulate the gut bacteria, fostering an environment suitable for digestion and nutritional absorption (Zhong et al., 2022). Animals with better gut health have increased growth rates, feed efficiency, and illness resistance. Additionally, the use of postbiotics as antibiotic replacements in livestock farming tackles issues with antibiotic resistance and promotes the development of healthy meat products (Rahman et al., 2022). By highlighting the significance of microbial populations in improving productivity and reducing the environmental impact of conventional agricultural operations, the application of postbiotics in both agriculture and animal health shows a holistic approach to sustainable practices (Manfredini et al., 2021).

## 7 Future perspectives

### 7.1 Advances in postbiotic production techniques

Future studies in animal models and clinical trials on humans are needed to determine the viability of postbiotics that support GI health (Malashree et al., 2019). Due to their equivalent positive benefits on health and low risk of introducing live bacteria, postbiotics are superior to probiotics, according to research in the field of biotics. Preterm newborns and those with weak immune systems will benefit the most from this in particular (Żółkiewicz et al., 2020). Necrotizing enterocolitis (NEC), a dreadful intestinal illness that affects premature or newborns with very low birth weights, is currently being treated with microbial metabolites. In addition, postbiotics are helpful in the treatment of diseases like multiple sclerosis and Alzheimer’s disease, for which there are currently no effective cures (Aggarwal et al., 2022). The field of postbiotics and numerous other related therapies have profited from a wealth of studies into microbiome-targeted diet and medication. There will be the emergence of new postbiotics, both inside and outside of the current classifications, testing the limits of science and regulation. Numerous metabolites will be obtained from novel sources that satisfy economic and environmental goals in order to fulfill a variety of compositional and functional areas in the microbiome (Sudhakaran et al., 2022). Postbiotics are extracts of dead bacteria and microorganisms that can strengthen the probiotics’ biological effects on the host. Because postbiotics are created when bacteria feed on prebiotics, eating a diet rich in probiotic and prebiotic foods may assist in guaranteeing that the gut has an adequate supply of these crucial nutrients. Clinical research is anticipated to shift in the future as more knowledge about postbiotics becomes available, focusing more on their composition as well as their bioactivity. Future studies should focus on identifying the link between the health effects of postbiotics and their unique mechanisms because postbiotics are continually changing (Thorakkattu et al., 2022).

### 7.2 Integration with precision medicine

Another cutting-edge topic for postbiotic research is the development of prototype “precision postbiotics” for effective therapeutic and preventative medicine. In contradistinction to a universal pharmacological intervention, precision medicine prioritizes the provision of medical care that is individualized and customized to suit the unique characteristics and requirements of each patient. Therefore, it is fascinating to build precise postbiotics for particular diseases in particular patient subgroups (Aggarwal et al., 2022). In healthy children, the use of postbiotics for a variety of illnesses or to promote general health has been shown to be effective; however, due to inconsistent findings in research looking into various causes of diarrhea, this practice should be used with caution, especially in children, in this situation. In these investigations, postbiotics exhibited favorable outcomes in treating diarrhea, in contrast to the findings for diarrhea in adults. Prebiotics were present in some of the items utilized in some research; therefore, it is important to proceed cautiously when evaluating their findings (Wegh et al., 2019).

### 7.3 Regulatory considerations

To the best of our knowledge, regulatory organizations have not yet developed a postbiotic framework or concept that specifically addresses the use of postbiotics in foods or dietary supplements. Postbiotic formulations for use in medicine or pharmaceuticals are subject to some regulatory regulations. The European Food Safety Authority (EFSA) regulates the necessities for food and updates them frequently in relation to the assessment of food safety in Europe. Contrarily, the European Pharmacopeia lays out clear rules that outline the maximum permitted levels of live microorganisms in pharmaceutical preparations and therapeutic goods (Thorakkattu et al., 2022). The Food and Drug Administration (FDA) in the United States has not made any specific statements regarding postbiotics. Because postbiotics can be manufactured under a variety of regulatory categories, the FDA will likely regulate postbiotics in accordance with the regulations that are specific to the regulatory category that has been selected for a product in development. The product must meet the requirements of the relevant regulatory category regarding its intended use, safety, and efficacy (Yelin et al., 2019). The FDA and EFSA’s probiotic regulatory frameworks, based on the generally recognized as safe (GRAS) and qualified presumption of safety (QPS) lists, do not apply to postbiotic preparations since they cannot contain live bacteria. Postbiotics are exempt from these frameworks. As a result, there appears to be a regulatory gap that allows for more freedom in product development and commercialization for postbiotic preparations than is strictly necessary to ensure that the postbiotic compounds themselves are not harmful. So, until the FDA and EFSA can develop a regulatory framework specifically for postbiotics, research is needed to determine a suitable set of safety and regulatory standards that should be applied to postbiotic preparations (Scott et al., 2022).

### 7.4 Commercialization opportunities

The biotics market is still evolving today. While consumers have a good understanding of probiotics, there still needs to be more awareness regarding prebiotics and postbiotics. Postbiotics will become more evident to industry professionals after being defined by the International Scientific Association of Probiotics and Prebiotics (ISAPP) in 2021 (Salminen et al., 2021). The postbiotics from *Lactobacillus* exhibit a variety of traits, including antibacterial and antioxidant activities, antibiofilm capabilities, as well as certain health advantages and medicinal uses for people; nevertheless, there are no commercially available postbiotics for food applications. Several barriers prevent the use of postbiotics in food technology, including safety concerns, insufficient *in vivo* and clinical trials, and commercially available postbiotics for human use (Moradi et al., 2020). Numerous postbiotics are currently commercially available that have uses besides food, but little is known about how to prepare them, how to analyze them, and what influences the production of each postbiotics compound. This lack of knowledge could hinder future research and widespread application in the food industry (Moradi et al., 2021). Selected soluble components of particular bacteria may develop into a group of biological strategies used by bacteria to treat a variety of ailments, but connecting science and business is extremely difficult (Aguilar-Toalá et al., 2018). Fermented foods have become the primary source of probiotics in commercial products with the purpose of promoting gut health, despite most consumers being unaware of the presence of microbial components. Nisin, a bacteriocin derived from postbiotics, has gained significant recognition for its widespread commercial application as a bio-preservative. The antimicrobial peptide, Nisin, is synthesized through the metabolic activities of a consortium of Gram-positive bacterial strains, specifically those belonging to the *Lactococcus* and *Streptococcus* genera. Nisin, synthesized by the bacterium *Lactococcus lactis*, has demonstrated notable efficacy in inhibiting biofilm formation and exerting antimicrobial activity against various oral pathogens. Consequently, it has promise as a potential postbiotic therapy for human usage, aiming to promote a healthy and beneficial oral microbiome (Scott et al., 2022).

## 8 Conclusion

This scholarly review article delves into the intricate realm of postbiotics, offering a comprehensive examination of their production, mechanisms of action, and diverse applications, particularly emphasizing their impact on human health and well-being. The examination of various production methodologies, including fermentation, enzymatic conversion, and synthetic biology methods, underscores the remarkable versatility of postbiotic formation. The study offers a road map for developing the sector by tackling the difficulties related to optimizing yields, improving stability, and ensuring safety. The information presented here not only expands our understanding of postbiotics but also reveals their potential to reshape the landscape of medicinal development and functional food ingredients. The insights offered in this review establish postbiotics as an important component in pursuing innovative and environmentally friendly options for human well-being as the scientific community continues to unravel the intricacies of the microbiome. Finally, the diverse perspective presented in this article contributes to the expanding body of research determining the future of postbiotics as transformative factor in the realms of health and nutrition.

## Author contributions

NP: Investigation, Methodology, Resources, Writing – original draft, Writing – review & editing. JP: Data curation, Investigation, Methodology, Validation, Writing – review & editing. SS: Formal analysis, Methodology, Resources, Supervision, Writing – review & editing. VY: Investigation, Software, Supervision, Validation, Writing – review & editing. CJ: Data curation, Formal analysis, Methodology, Project administration, Writing – review & editing. AnP: Conceptualization, Formal analysis, Investigation, Supervision, Writing – review & editing. DP: Data curation, Investigation, Project administration, Software, Writing – review & editing. DS: Funding acquisition, Methodology, Supervision, Visualization, Writing – review & editing. AsP: Conceptualization, Project administration, Supervision, Visualization, Writing – original draft, Writing – review & editing.
